# Genome Sequence of *Pseudomonas aeruginosa* PA45, a Highly Virulent Strain Isolated from a Patient with Bloodstream Infection

**DOI:** 10.1128/genomeA.00289-13

**Published:** 2013-05-23

**Authors:** Nicola Segata, Annalisa Ballarini, Olivier Jousson

**Affiliations:** Centre for Integrative Biology, University of Trento, Trento, Italy

## Abstract

*Pseudomonas aeruginosa* is a ubiquitous opportunistic pathogen causing a broad range of infections in humans. We provide the draft genome sequence of the recently identified and highly virulent *P. aeruginosa* PA45 strain. Its 6.6-Mb genome contains 6,822 genes, including an unparalleled number of virulence genes, which might explain its aggressive phenotype.

## GENOME ANNOUNCEMENT

*Pseudomonas aeruginosa* is a ubiquitous environmental bacterium and an opportunistic pathogen causing acute and chronic infections in the urinary tract, blood, skin, and lungs of immunocompromised patients. *P. aeruginosa* strains and virulent variants causing severe human chronic infections, e.g., to the lungs of cystic fibrosis patients, have been extensively studied, while strains causing acute human infections need further investigation, primarily to define their pathogenic potential. In a previous study (1), we characterized the virulence phenotype of a collection of clinical *P. aeruginosa* strains that cause acute infections and identified the highly aggressive PA45 strain, a clinical isolate that caused a severe acute bloodstream infection in a hospitalized oncology patient who was undergoing chemotherapy treatment. Here, we report the draft genome sequence of the *P. aeruginosa* PA45 strain, thus providing new information about the genomic determinants underlying its highly aggressive phenotype.

The *P. aeruginosa* PA45 genome was sequenced using the 454 GS-FLX (Roche) platform, generating 854,562 raw reads, for a total of 332,906,173 bp with 50× average coverage (398 average read length). Using the Newbler 2.8 assembler, reads were assembled into 124 contigs with an N_50_ size of 220,690. Open reading frames, rRNAs, and tRNAs were identified and annotated using the pipeline provided by the Integrated Microbial Genomes-Expert Review (2). The genome is 6,615,955 bp long, exceeding the size of 9 of the 12 *P. aeruginosa* genomes available in the IMG database (3) (average 6,474,902 bp; standard deviation [SD], 226,090). The average G+C content is 66.3%, and 6,822 genes, including 3 rRNA and 58 tRNA genes, were identified. The annotation procedure successfully profiled 82.1% of genes with a specific function, 78.9% with a COG assignment, 48.77% with a KEGG orthology family, and 84.3% with Pfam domains. Of the 3,832 core genes shared by all 12 *P. aeruginosa* sequenced strains (4), 3,822 (99.7%) genes are found also in PA45, confirming the completeness of the core genome assembly and the presence of all functional modules characterizing this species. Compared to the accessory genome, which is unique to each of the already-sequenced strains, PA45 was found to be closely related to *P. aeruginosa* C3719 (GenBank accession no. NZ_AAKV00000000), carrying 74.4% of its unique genes (e-value, <1e – 20; length, >200 bp), whereas *P. aeruginosa* NCGM2.S1 (GenBank accession no. AP012280) and *P. aeruginosa* 39016 (GenBank accession no. AEEX00000000) had the smallest number of overlapping strain-specific genes (20.8% and 23.0%, respectively). Compared to the other 12 *P. aeruginosa* strains, PA45 comprises the highest number of virulence genes (829) in its genome, according to the BLASTx translated search we performed against the Virulence Factor database (DB) (5). Focusing on the pathogenicity islands PAPI-1 and PAPI-2 carried by the highly virulent PA14 strain (GenBank accession no. NC_008463), 82 out of 113 PAPI-1 genes and 16 out of 19 PAPI-2 genes were found in PA45 (e-value, <1e − 20). The unprecedented high abundance of virulence gene homologues carried by the PA45 strain correlates well with its highly aggressive phenotype.

### Nucleotide sequence accession numbers.

This Whole-Genome Shotgun project has been deposited at DDBJ/EMBL/GenBank under the accession no. APMD00000000. The version described in this paper is the first version, accession no. APMD01000000.

## References

[1] Janjua H Segata N Bernabò P Tamburini S Ellen A Jousson O 2012 Clinical populations of *Pseudomonas aeruginosa* isolated from acute infections show a high virulence range partially correlated with population structure and virulence gene expression. Microbiology 158:2089–2098 22556359 10.1099/mic.0.056689-0

[2] Markowitz VM Mavromatis K Ivanova NN Chen IMA Chu K Kyrpides NC 2009 IMG ER: a system for microbial genome annotation expert review and curation. Bioinformatics 25:2271–2278 19561336 10.1093/bioinformatics/btp393

[3] Markowitz VM Chen IM Palaniappan K Chu K Szeto E Grechkin Y Ratner A Jacob B Huang J Williams P Huntemann M Anderson I Mavromatis K Ivanova NN Kyrpides NC 2012 IMG: the Integrated Microbial Genomes database and comparative analysis system. Nucleic Acids Res. 40:D115–D122 22194640 10.1093/nar/gkr1044 PMC3245086

[4] Segata N Huttenhower C 2011 Toward an efficient method of identifying core genes for evolutionary and functional microbial phylogenies. PLoS One 6:e24704 21931822 10.1371/journal.pone.0024704 PMC3171473

[5] Chen L Xiong Z Sun L Yang J Jin Q 2012 VFDB 2012 update: toward the genetic diversity and molecular evolution of bacterial virulence factors. Nucleic Acids Res. 40:D641–D645 22067448 10.1093/nar/gkr989 PMC3245122

